# Universal Health Coverage and Health Equity in West Africa: Tracking Progress Toward SDG 3.8 in Ghana and Liberia

**DOI:** 10.1002/puh2.70280

**Published:** 2026-05-27

**Authors:** Samuel Asamoah, Michael Sarfo, Olanrewaju Lawal, Sandra Konadu Bonnah, Michael Snowden, Jamie P. Halsall, Godness Kye Biney, Angwach Abrham Asnake

**Affiliations:** ^1^ Kumasi Centre for Collaborative Research in Tropical Medicine Kumasi Ghana; ^2^ School of Human and Health Sciences University of Huddersfield Huddersfield UK; ^3^ Department of Health Promotion and Disease Prevention University of Nebraska Medical Center Omaha Nebraska USA; ^4^ Department of Biostatistics and Epidemiology School of Public Health and Health Sciences University of Massachusetts Amherst Amherst Massachusetts USA; ^5^ Department of Epidemiology and Biostatistics School of Public Health College of Health Sciences and Medicine Wolaita Sodo University Wolaita Sodo Ethiopia

**Keywords:** Demographic and Health Survey, Ghana, health equity, health inequality, health services coverage, Liberia, maternal health, Sustainable Development Goal (SDG) 3.8, universal health coverage

## Abstract

**Background:**

Achieving universal health coverage (UHC) remains a key priority for many low‐ and middle‐income countries, yet equity in access continues to be a significant challenge. This study examines the level and distribution of health service coverage in Ghana and Liberia, with a particular focus on wealth‐ and residence‐based disparities, to assess progress toward UHC and Sustainable Development Goal (SDG) 3.8.

**Methods:**

A cross‐sectional analysis was conducted using the most recent Demographic and Health Survey (DHS) data from Ghana and Liberia. Key indicators related to maternal health, health promotion, environmental health and infectious disease treatment were assessed. Composite indices for protection and treatment coverage were computed. Inequality was measured using the slope index of inequality (SII), relative index of inequality (RII), absolute difference index (ADI) and the urban–rural coverage ratio.

**Results:**

Overall, Ghana demonstrated higher coverage in preventive services such as antenatal care (90.6%) and improved drinking water (91.8%), whereas Liberia showed slightly higher treatment coverage for malaria and respiratory infections. However, both countries exhibited substantial pro‐rich inequities in skilled birth attendance and insecticide‐treated net (ITN) use. In Ghana, a rural advantage was observed in some indicators, likely due to targeted outreach through the Community‐Based Health Planning and Services (CHPS) initiative, whereas Liberia displayed mixed urban–rural disparities. The composite coverage index (CCI) was higher in Liberia (77.9%) than in Ghana (63.3%), reflecting recent donor‐supported health system investments in Liberia post‐Ebola.

**Conclusion:**

Despite moderate national coverage levels, significant socio‐economic and geographic disparities persist in both countries. Although targeted policies, such as Ghana's National Health Insurance Scheme (NHIS) maternal exemption and Liberia's post‐crisis recovery programs, have contributed to localized improvements, inequities in access to essential health services remain. Addressing these disparities is critical for both countries to make meaningful progress toward equitable UHC.

## Graphical abstract

Despite moderate national progress toward universal health coverage in Ghana and Liberia, significant wealth‐ and location‐based inequities persist. Preventive services are relatively strong, but access to essential treatments remains low, highlighting the need for targeted, equity‐focused health policies to achieve SDG 3.8.

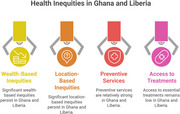

## 1 Introduction

Universal health coverage (UHC) is described as ensuring equitable access to quality healthcare for all individuals in need, without imposing financial burdens on those receiving care [1]. Equity appears fundamental to the pursuit of UHC; for example, the World Health Report 2008 characterises universal coverage reforms as initiatives that ensure health systems promote health equity, social justice and the cessation of exclusion, primarily by advancing universal access and social health protection [2]; the World Health Organization (WHO) posits that equity is a key intermediate objective of UHC [3]; and the WHO Consultative Group on Equity and UHC encourages nations to prioritise fairness, equity and health rights in their policymaking [4]. Nonetheless, fairness does not inherently result from the execution of UHC programs. Conversely, the pursuit of UHC entails trade‐offs that may not benefit vulnerable populations, and certain policies implemented under the guise of UHC could exacerbate inequalities [5, 6, 7, 8]. Progress toward UHC is monitored globally through Sustainable Development Goal (SDG) Target 3.8, which aims to achieve UHC, including financial risk protection, access to quality essential healthcare services and access to safe, effective, quality and affordable essential medicines and vaccines for all [9, 10]. This target emphasizes that service expansion must not come at the cost of financial hardship for the user. However, certain policies implemented under the guise of UHC could exacerbate inequalities. For example, social health insurance schemes in some African nations have been criticized for primarily covering formal sector workers, effectively subsidizing a privileged urban minority while leaving the poorest populations, often in the informal sector, without adequate protection [7]. Furthermore, focusing on high‐end hospital care rather than primary health centres can lead to elite capture, where limited public funds benefit those who already have the best access [7, 8].

Numerous low‐ and middle‐income countries (LMICs) have initiated health system reforms to attain UHC [11, 12]. These changes aim to establish or enhance public healthcare funding systems that consolidate resources from various prepaid financing sources, serving as alternatives to out‐of‐pocket expenditures [12, 13, 14]. The policy purpose of UHC is to guarantee that all citizens of a nation have sufficient coverage through prepaid finance schemes and have access to necessary, high‐quality health treatments [12, 14, 15]. Over the past two decades, most countries in Africa have prioritized UHC. Notwithstanding this, the shift to UHC in Africa is perceived as gradual, with certain nations encountering stagnation [16].

It has been demonstrated through study that, on average, it would take a decade to attain moderate Universal health coverage, although progress differs substantially across populations and dimensions of UHC [17]. However, the movement toward UHC dimensions of population, service and financial coverage has been inconsistent and sluggish [18]. Population and service coverage of 60%–90% are observed in certain countries, including Tunisia [19], Rwanda and Morocco [20, 21]. Conversely, Ghana [22], Gabon [23], Nigeria [24], Kenya [25], Zambia [26] and Senegal [17] exhibit coverage between 20% and 500% depending on the indicator and year of assessment. In the wake of recent crises, such as protracted civil conflicts and the Ebola outbreak, the WHO's UHC Service Coverage Index [27], which monitors national advancements toward SDG 3.8.1, indicates that as of 2019, only 39% of Sierra Leone has attained UHC, whereas Liberia is at 42%. Numerous studies have evaluated the efficacy of the reforms and Africa's advancement toward UHC concerning the three dimensions defined by the WHO [28, 29]. Despite efforts to achieve SDG 3 (Good Health and Well‐being), Ghana and Liberia face significant challenges in reaching UHC, particularly in primary healthcare access and health equity. This study seeks to address the knowledge gap regarding UHC by evaluating access to primary healthcare, health education and population‐level interventions in both Ghana and Liberia, utilising secondary data from the Demographic Health Survey (DHS) for each country and aiming to identify key factors such as health service coverage and inequalities hindering progress toward SDG 3.8 in both countries in a post‐pandemic era.

## 2 Methodology

### 2.1 Study Design

A cross‐sectional design using secondary data from the DHS was employed in this study to evaluate access to primary healthcare, health education and population‐level interventions in two West African countries: Ghana and Liberia.

### 2.2 Source of Data

The DHS data collected between 2018 and 2020 were used for this study. The DHS program is a population‐based survey conducted in many countries around the world using a uniform protocol, adapted to meet local uniqueness, to collect data that assist nations in monitoring, analysing and evaluating public health programs and interventions. Health demographic data, including maternal and child health, fertility, family planning utilization, morbidity and mortality, are gathered in this survey. The dataset is accessible through the official DHS website, https://dhsprogram.com.

### 2.3 Study Settings and Population

Two developing countries in sub‐Saharan Africa were selected because of their similar characteristics. Both Ghana and Liberia are developing nations in West Africa with relatively fragile health systems [30, 31]. This fragility is marked by critical shortages in the health workforce and infrastructure deficits. Although Ghana has maintained relative stability, Liberia's health system is still in a phase of recovery from protracted civil conflicts and the 2014–2016 Ebola outbreak, which severely decimated its medical workforce and disrupted trust in formal healthcare delivery. Liberia is recovering from civil conflicts that displaced almost half of its population. Comparatively, Ghana has had no ethnopolitical conflict in recent times, making it suitable to compare the progress of these countries toward the 2030 milestone of UHC.

All women of reproductive age between 15 and 49 years, residing in all regions of Ghana and Liberia and included in the individual record (IR) dataset, were studied.

### 2.4 Variables and Indicators Selection

Variables representing the UHC indicators for maternal and child health, health promotion and environmental health were selected. These include antenatal care (ANC) coverage, health promotion coverage, sanitation coverage, access to improved drinking water, access to antenatal malaria prophylaxis, malaria prevention interventions and treatment for malaria and respiratory illnesses. The sanitation variable was derived from the variable (V116)—the type of toilet facility used. The original variable, V113, categorized the source of drinking water into safe and unsafe sources to create the variable for improved drinking water. The number of antenatal visits was grouped into more than four visits or fewer. The treatment for malaria variable was derived from all children receiving artemisinin combination therapy, compared to those receiving other therapies or no treatment for fever. Treatment for respiratory infections was obtained from participants receiving medical care for cough and fever. The health promotion variable was selected from the original variable, S501—participants who have heard or been educated about malaria in the past 6 months via all media.

### 2.5 Health Services Coverage

To align with previous studies [32, 33] and ensure effective comparison between the two countries, a composite coverage index (CCI) was computed for the countries from six interventions from three key health specialties: maternal care, health promotion and infectious disease case management:

$$\mathrm{CCI}=\frac{1}{3}\left(\frac{\mathrm{ANC}+\mathrm{SBA}+\mathrm{IPT}}{3}+\mathrm{HPM}+\frac{\mathrm{RTI}+\mathrm{ACT}}{2}\right)$$

where ANC represents antenatal care coverage, SBA represents skilled birth attendants, IPT represents intermittent treatment for malaria in pregnant women, HPM represents health promotion coverage, RTI represents respiratory tract infection treatment and artemisinin‐combined therapy for malaria treatment.

Random meta‐analysis was performed to estimate the mean proportion of the protection coverage, and treatment coverage indices based on nine indicators and two treatment indicators, respectively. In all computations and analyses, the survey design was taken into consideration.

### 2.6 Health Inequities Analyses

Slope index inequality (SII) and relative index inequality (RII) indices were used (from the healthequal package in R) to estimate wealth‐based inequalities. The SII expresses the absolute difference in coverage percentage points between the extremes of the wealth quintile and gives the actual effort that will be needed to close the gap in inequalities. However, the RII gives the idea of the degree of inequity by estimating the ratio of intervention coverage for poor and wealthy households. Residential‐based inequalities were also estimated using the absolute difference (ADI), and the urban–rural coverage ratio was performed to find the inequalities among rural and urban dwellers.

### 2.7 Statistical Analysis

Data preparation, cleaning and analysis were performed using R version 4.3. The mean proportion of the health indicators was used to estimate the coverage for both countries. Using the meta package in R, a random meta‐analysis was performed to estimate the mean coverage for protection and treatment indices on the basis of seven intervention indicators and two treatment indicators, respectively. The health equal package in R 4.3 software was also used to measure health service coverage inequities.

### 2.8 Ethical Consideration

We used a secondary dataset that is freely available to the public from the DHS program; therefore, no ethical approval was requested. The dataset is anonymized, and more details regarding its ethical standards are available at http://goo.gl/ny8T6X.

## 3 Results

### 3.1 Socio‐Economic Distribution

**FIGURE 1:**
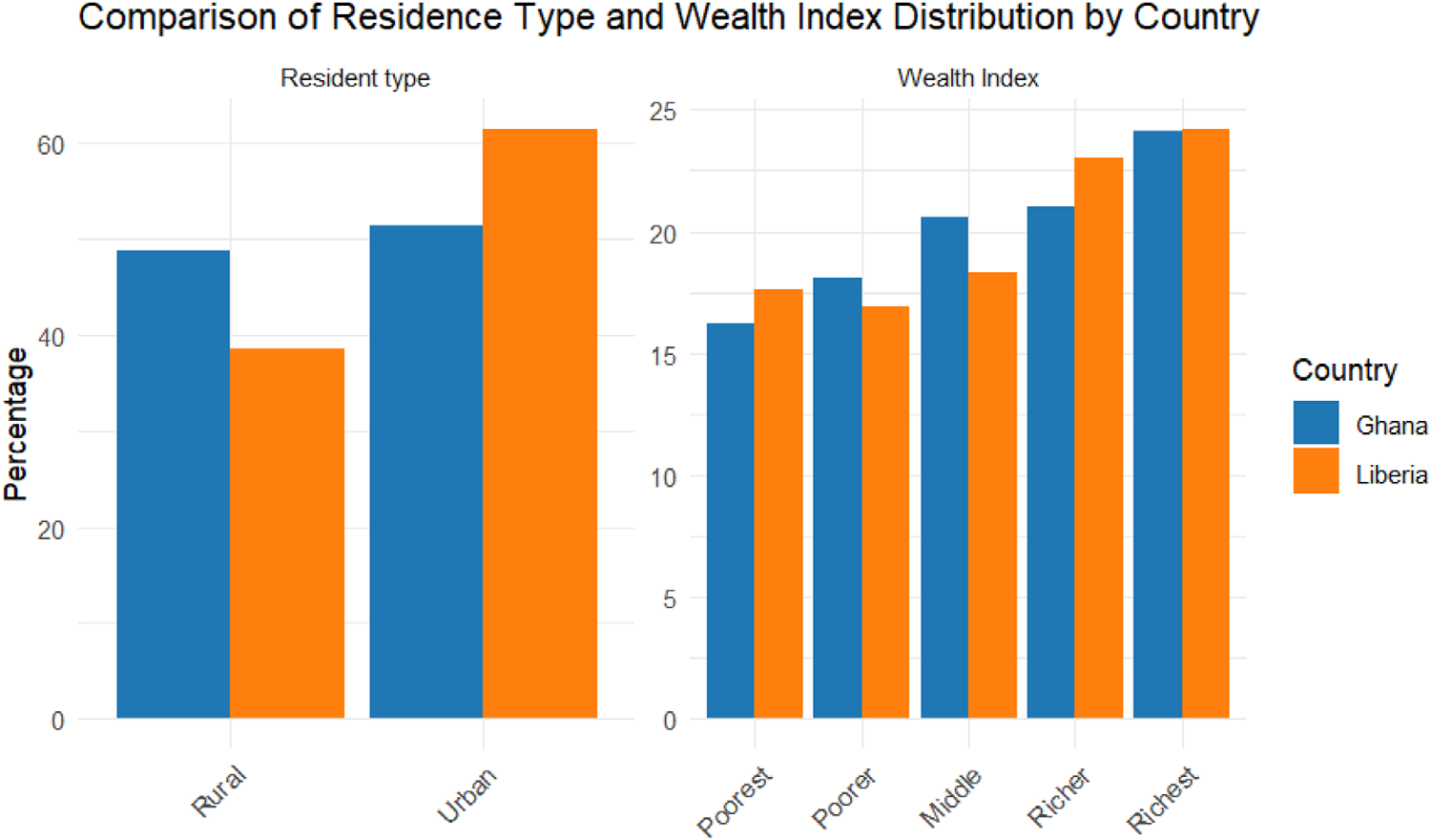
Bar plots showing the residence and wealth status of participants involved in this study.

A total weighted sample of 9694 women of reproductive age (15–49 years old) living in Ghana and Liberia was studied (see Table 1 and Figure 1). About 5181 Ghanaian women of the target age group were included, representing 53% of the total participants, with the remaining from Liberia. Most of the Ghanaian respondents (51%) were urban dwellers compared to 63% in the Liberian sample. Of all the respondents, 16% and 17% live in extreme poverty in Ghana and Liberia, respectively, with most falling into the middle class. Both countries have 24% of the participants at the top of the wealth strata.

**TABLE 1:** Socio‐economic distribution.

Variable	Category	Ghana (*n*) and (%)	Liberia (*n*) and (%)
Wealth index	Poorest	839 (16.2)	794 (17.6)
Wealth index	Poorer	940 (18.1)	764 (16.9)
Wealth index	Middle	1069 (20.6)	827 (18.3)
Wealth index	Richer	1087 (21.0)	1037 (23.0)
Wealth index	Richest	1247 (24.1)	1091 (24.2)
Residence	Urban	2657 (51.3)	2771 (61.4)
Residence	Rural	2524 (48.7)	1742 (38.6)

*Note:* This table represents the financial status and residence of participants from both countries.

### 3.2 Health Services Coverage

The overall mean percentage of protection intervention coverage varies between the two countries. Coverage is relatively higher in Ghana, with approximately 68% (64.8%–70.8%), compared to about 66% (62.5%–69.7%) in Liberia. Nationwide coverage of the specific preventive interventions also differs, with Ghana performing well in nearly all indicators (see Table 2). However, coverage for malaria prophylaxis is similar in both Ghana and Liberia (see Table 2). Treatment coverage for prevalent infectious diseases in the region shows slight variation, with Liberia having a slight advantage. Liberia's national treatment coverage is 61.2% (59.8%–62.5%) compared to 60.1% (59.4%–60.8%) in Ghana. The trends in specific indicators are similar, especially in malaria treatment. Artemisinin combination therapy for malaria is markedly low in both countries, with Liberia at about 48% (95% CI: 44.1%–52.5%) and Ghana at approximately 40% (95% CI: 35.6%–44.1%). Treatment coverage for respiratory infections is almost identical in both countries: 42% (37.7%–46.3%) in Ghana and 42.5% (37.9%–47%) in Liberia. The CCI for maternal care, health promotion and case treatment stands at 63.3% (61.1%–65.4%) in Ghana and 77.9% (75.2%–80.6%) in Liberia.

**TABLE 2:** Health services coverage in both countries.

Indicator	Ghana	Liberia
**Prevention**		
Skilled birth attendant	41.7% (39.9%–43.4%)	32.8% (30.5%–35%)
Antenatal care	90.6% (88.7%–92.5%)	84.6% (81.7%–87.6%)
Access to insecticide‐treated net	80% (78.2%–81.8%)	76.9% (73.4%–80.3%)
Access to health promotion/education	59% (56.1%–61.8%)	49.8% (46.6%–53%)
Improved drinking water	91.8% (89.1%–94.4%)	86.6% (83%–90.2%)
Malaria prophylaxis for pregnant women	87.1% (85.3%–89%)	86.5% (83.8%–89.2%)
Sanitation	73.2% (69.7%–76.6%)	53.1% (47.8%–58.3%)
**Treatment**		
Artemisinin‐combined therapy for malaria	39.9% (35.6%–44.1%)	48.3% (44.1%–52.5%)
Respiratory infection treatment	42% (37.7%–46.3%)	42.5% (37.9%–47%)
**Composite indices**		
Protection coverage index	67.9% (64.8%–70.8%)	66.2% (62.5%–69.7%)
Treatment coverage index	60.1% (59.4%–60.8%)	61.2% (59.8%–62.5%)
Composite coverage index	63.3% (61.1%–65.4%)	77.9% (75.2%–80.6%)

*Note:* The mean percentage of healthcare indicators representing coverage in the two countries. A higher percentage indicates higher coverage for all manner of populations.

### 3.3 Health Inequity Analyses

#### 3.3.1 Wealth‐Based Inequity Measurement

**FIGURE 2:**
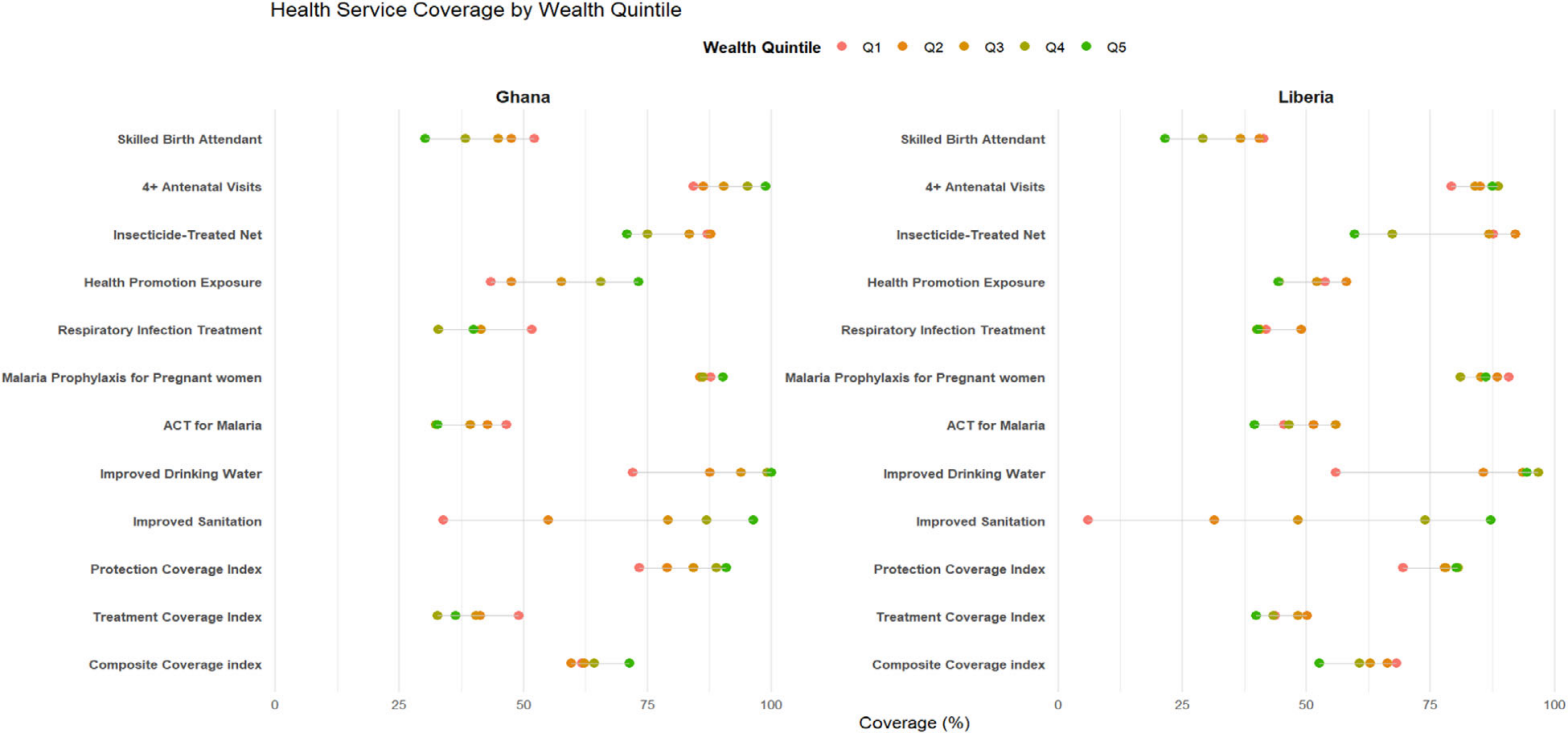
This dot plot represents the proportion of coverage within the wealth quintile. The range of measures was from Q1 (poorest quintile) to Q5 (wealthiest quintile). ACT, artemisinin‐combined therapy for malaria treatment.

In Ghana, a pro‐poor inequity was observed in ANC services, although the disparity was not statistically significant in either country. The poorest group was 36% more likely to access at least four ANC visits compared to the wealthiest in Ghana (RII = 0.64, SII = −44.93), whereas in Liberia, the wealthiest women had a 19% advantage (RII = 1.19, SII = 17.6) (See Table 3 and Figure 2). Access to SBA follows a similar pattern in both countries, with the wealthiest pregnant women having access to a qualified skilled birth attendant compared to the poorest. This difference was statistically significant, with nearly 2.5 times the advantage in Liberia (RII = 2.48, 95% CI: 1.72–3.58) and about 94% greater access among the richest in Ghana (RII = 1.94, 95% CI: 1.46–2.59).

**TABLE 3:** Wealth‐based inequity of both countries.

	Ghana		Liberia	
Indicator	Slope index inequality (95% CI)	Relative index inequality (95% CI)	Slope index inequality (95% CI)	Relative index inequality (95% CI)
Skilled birth attendant	66.5 (37.96–95.04)	1.94 (1.46–2.59)	90.93 (54.39–127.48)	2.48 (1.72–3.58)
Antenatal care	−44.93 (−119.53 to 29.66)	0.64 (0.3–1.35)	17.57 (−66.33 to 101.46)	1.19 (0.52–2.76)
Insecticide‐treated net	111.43 (66.37–156.49)	3.05 (1.94–4.78)	280.1 (194.9–365.3)	16.46 (7.02–38.59)
Health promotion	−87.24 (−125.58 to 48.9)	0.42 (0.28–0.61)	93.27 (45.15–141.39)	2.54 (1.57–4.11)
Seeking care for respiratory infections	144.68 (83.23–206.14)	4.25 (2.3–7.86)	78.82 (9.49–148.14)	2.2 (1.1–4.4)
Malaria prophylaxis for pregnant women	53.61 (0.51–106.7)	1.71 (1.01–2.91)	115.73 (19.24–212.23)	3.18 (1.21–8.35)
Combined artemisinin for malaria	23.86 (−46.48 to 94.2)	1.27 (0.63–2.57)	74.73 (8.07–141.4)	2.11 (1.08–4.11)
Improved drinking water	−151.89 (−267.63 to 36.15)	0.22 (0.07–0.7)	−173.7 (−290.6 to −56.81)	0.18 (0.05–0.57)
Sanitation	−252.06 (−326.52 to 177.61)	0.08 (0.04–0.17)	−238.05 (−337.92 to −138.17)	0.09 (0.03–0.25)
Protection coverage index	−70.63 (−100.35 to 40.91)	0.49 (0.37–0.66)	0.54 (−30.85 to 31.93)	1.01 (0.73–1.38)
Treatment coverage index	83.86 (33.33–134.4)	2.31 (1.4–3.83)	76.49 (16.73–136.25)	2.15 (1.18–3.91)
Combined coverage index	−3.23 (−33.38 to 26.93)	0.97 (0.72–1.31)	84.97 (45.08–124.87)	2.34 (1.57–3.49)

*Note:* A representation of wealth‐based inequity of access to health services and interventions in Ghana and Liberia. A positive slope inequality index (SII) denotes pro‐wealth inequity. The magnitude shows the absolute margin between the richest and the poorest. The relative inequality index measures the ratio of inequality. RII = 1 means equity, whereas >1 means pro‐wealth and <1 means pro‐poor inequity.

Insecticide‐treated net (ITN) use showed the most inequality in Liberia, where the wealthy were 16.5 times more likely to possess insecticide–treated nets (RII = 16.46, 95% CI: 7.02–38.59). A similar pattern appeared in Ghana, with the rich being three times more likely to have ITNS than the poorest (RII = 3.05, 95% CI: 1.94–4.78).

Significant wealth‐based disparities were also observed in access to improved drinking water and sanitation in both countries. A rural advantage was noted in these indicators, though marginal. In Ghana, the lowest wealth group had a 22% and 8% advantage in accessing quality drinking water and improved sanitation, respectively, compared to the richest group. Similarly, in Liberia, access to water and sanitation favoured the poorest class, with 18% and 9% advantages, respectively.

Varying disparities were seen in access to health promotion or education. In Ghana, the poorest had a 42% advantage, whereas in Liberia, health promotion favoured the richest group, with a relative inequality index of 2.54.

Protection coverage also showed significant inequality in Ghana (RII = 0.49, 95% CI: 0.37–0.66), with the poor experiencing a 49% advantage. In Liberia, the overall protection service coverage was nearly equitable, with the wealthy having just a 1% advantage and a gap of 0.0.54. Treatment coverage favoured the wealthy in both countries, with a disparity of approximately twofold in favour of the rich (see Table 3).

#### 3.3.2 Residential‐Based Inequity

Table 4 presents the urban–rural health coverage inequality across Ghana and Liberia. In Ghana, although access to skilled birth attendants is generally low, urban dwellers have about 13% (relative ratio index [RRI] = 1.13, 95% CI: 1.03–1.22) more access to skilled birth attendants with a marginal gap point of about 5 between rural dwellers (ADI = 4.98, 95% CI: 1.48–8.49). An urban dweller in Ghana is 31% more likely to receive care for acute respiratory infections (ARI) than a rural resident (RRI = 1.31, 95% CI: 1.05–1.56). In addition, ITN coverage is higher among urban areas than rural areas (ADI = 6.10, 95% CI: 2.09–10.11), and the overall treatment coverage also favours urban areas (ADI = 7.14, 95% CI: 0.26–14.03). Rural households, on the other hand, have access to improved sanitation (ADI = −13.80, 95% CI: −20.87 to −6.74) and overall protective interventions coverage, quality drinking water (ADI = −7.52, 95% CI: −12.42 to −2.6) and preventive interventions coverage (ADI = −3.53, 95% CI: −6.01 to −1.05). However, urban–rural inequalities seen in ANC coverage, health promotion, malaria prophylaxis in pregnancy (IPT), ACT for malaria and improved drinking water coverage were not statistically significant.

**TABLE 4:** Urban–rural health service coverage inequality: Ghana and Liberia.

	Ghana				Liberia			
Indicators	Urban coverage %	Rural coverage %	Absolute difference index (95% CI)	Relative ratio index (RRI) (95% CI)	Urban coverage %	Rural coverage %	Absolute difference index (95% CI)	Relative ratio index (RRI) (95% CI)
Skilled birth attendant	44.7	39.7	4.98 (1.48–8.49)^a^	1.13 (1.03–1.22)^a^	34.3	31.8	2.47 (−2.05 to 6.85)	1.08 (0.93–1.22)
Antenatal care	89.3	91.5	−2.26 (−6.45 to 1.93)	0.98 (0.93–1.02)	86.0	83.1	2.9 (−3.03 to 8.84)	1.03 (0.96–1.11)
ITN	83.8	77.7	6.10 (2.09–10.11)^a^	1.08 (1.03–1.13)^a^	81.8	73.9	7.88 (0.32–15.44)^a^	1.11 (1.00–1.21)
Health promotion	55.8	60.9	−5.19 (−10.80 to 0.41)	0.91 (0.83–1.00)	50.2	49.6	0.62 (−6.65 to 7.89)	1.01 (0.87–1.16)
Treatment for respiratory infection	48.5	37.1	11.36 (3.10–19.62)^a^	1.31 (1.05–1.56)^a^	45.5	40.2	5.23 (−3.92 to 14.39)	1.13 (0.89–1.37)
Malaria prophylaxis for pregnant women	86.3	87.7	−1.31 (−4.89 to 2.28)	0.99 (0.94–1.03)	85.9	86.9	−1.02 (−6.90 to 4.85)	0.99 (0.92–1.06)
Artemisinin‐combined therapy for malaria	41.5	38.6	2.92 (−5.83 to 11.67)	1.08 (0.84–1.31)	50.9	46.4	4.53 (−3.88 to 12.94)	1.10 (0.91–1.29)
Improved drinking water	90.2	92.8	−2.54 (−7.43 to 2.35)	0.97 (0.92–1.02)	84.3	87.9	−3.60 (−10.51 to 3.31)	0.96 (0.88–1.04)
Sanitation	64.7	78.5	−13.80 (−20.87 to 6.74)^a^	0.82 (0.74–0.91)^a^	48.1	56.1	−7.97 (−18.88 to 2.93)	0.86 (0.67–1.04)
Protection index	80.9	84.4	−3.53 (−6.01 to 1.05)^a^	0.96 (0.93–0.99)^a^	76.8	77.0	−0.22 (−2.70 to 2.26)	1.00 (0.97–1.03)
Treatment index	45.0	37.9	7.14 (0.26–14.03)^a^	1.19 (0.99–1.38)	48.2	43.3	4.88 (−2.51 to 12.28)	1.11 (0.94–1.29)
Composite coverage index	63.3	63.2	0.10 (−3.82 to 4.01)	1.00 (0.94–1.06)	64.8	61.2	3.57 (−1.29 to −8.44)	1.06 (0.98–1.14)

*Note:* This table presents the absolute difference index (ADI) and relative ratio index (RRI) to measure residential type‐based health intervention inequity. The absolute difference measures the magnitude of inequality, and the relative ratio index (RRI) measures the strength of urban–rural inequity. Negative ADI shows a rural advantage, whereas positive ADI shows urban advantage. RRI >1 represents higher urban strength, and <1 shows higher rural strength.

^a^Statistically significant inequalities.

**FIGURE 3:**
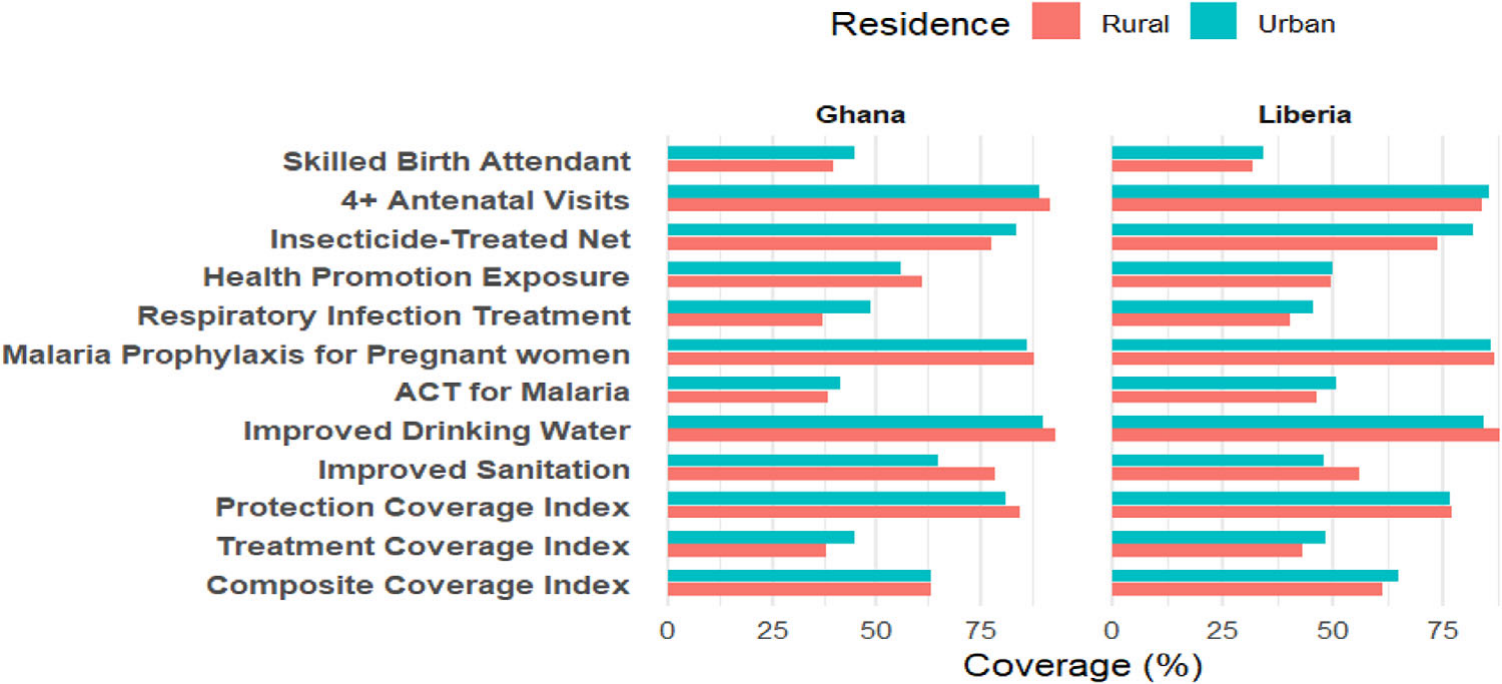
A side‐by‐side bar chart represents the percentage of health interventions coverage between urban and rural dwellers in both countries. ACT, artemisinin‐combined therapy for malaria treatment.

In Liberia, urban–rural inequalities in health service coverage were generally limited across most indicators. Among the indicators examined, ITN use was the only service demonstrating statistically significant inequality favouring urban populations. Urban residents had higher ITN coverage compared with rural residents (81.8% vs. 73.9%), with an ADI of 7.88 (95% CI: 0.32–15.44) and an RRI of 1.11 (95% CI: 1.00–1.21). Figure 3 illustrates the urban–rural health services coverage in Ghana and Liberia.

## 4 Discussion

This study assessed health service coverage and inequalities in Ghana and Liberia using DHS data. Although both countries show moderate levels of overall health service coverage, substantial disparities remain, particularly along wealth and residential lines, undermining the equity goals central to UHC.

We found relatively high coverage of preventive services such as ANC and access to improved drinking water across both countries. This finding aligns well with a study conducted by Barros et al. which asserts that these services are often regarded as essential services that receive prioritization in maternal and child health programs. Ghana's impressive performance in preventive care services, such as its protection coverage index of 67.9% compared to Liberia, may reflect longstanding investments in primary healthcare infrastructure and health promotional campaigns under the support of the National Health Insurance Scheme (NHIS) and trained healthcare experts who go to the length and breadth to offer health service to people even in the hinterlands [34, 35]. On the contrary, Liberia's relatively high treatment coverage index (61.2%) likely reflects substantial post‑Ebola international aid and health systems strengthening efforts, including major donor investments in workforce training, infrastructure, supply chains and governance under the country's 2015–2021 investment plan. Indeed, studies document treatment for childhood fever (malaria) improving from 45% in 2007 to 85% by 2019–2020, with rapid service rebound following the Ebola outbreak [31, 36].

Interestingly, our study found that coverage for essential treatments, particularly artemisinin‐based therapy for malaria and the management of respiratory infections, remains low in both countries. Specifically, the low ACT coverage observed in our findings aligns with previous studies [37, 38], which highlight the role of economic inequalities, where wealthier families are more likely to access treatment than poorer households. This disparity can be attributed to factors such as fragmented supply chains, affordability constraints, gaps in provider knowledge and concerns regarding drug quality [37, 39]. Although less frequently studied, similar patterns appear to exist in the treatment of pneumonia, where evidence from Ghana links maternal financial insecurity and limited access to services with higher rates of ARI in children and a lower likelihood of receiving timely treatment in disadvantaged populations [40]. This finding is particularly significant in West Africa, where malaria infections and respiratory diseases remain highly prevalent [41]. The persistently low coverage of ACT undermines national efforts to achieve SDG 3 by 2030 and the broader goal of eliminating malaria as a public health threat. Beyond economic and logistical barriers, the deep‐seated role of traditional medicine serves as a significant contextual driver for the low uptake of ACT and respiratory treatments. In both Ghana and Liberia, traditional healers are often the first point of care for childhood fevers due to their cultural proximity and perceived affordability compared to formal clinical settings [42, 43]. This reliance on traditional remedies can delay the seeking of evidence‐based therapies, effectively masking the true demand for ACTs. Furthermore, the persistent fragility of these systems, characterized by workforce shortages and disrupted trust following the Ebola crisis in Liberia, continues to deter disadvantaged populations from utilizing formal health services even when coverage technically exists.

Our strong findings of pro‐rich inequity in SBA and ITN use align with prior studies from Ghana, where SBA coverage is significantly higher among wealthier women, largely due to differences in health insurance enrolment, educational attainment and urban [44, 45].

Similarly, ANC services, though increasing in overall coverage, remain skewed toward socio‐economic advantage, with the wealthiest and most educated women showing consistently higher utilization [46, 47]. However, policies, such as Ghana's NHIS maternal exemption introduced in 2008 and the expansion of the Community‐Based Health Planning and Services (CHPS) initiative, have demonstrably reduced financial and geographic barriers, especially in rural and underserved areas, contributing to more equitable ANC coverage among poorer groups [48]. In Liberia, the context is shaped by post‐conflict reconstruction and the aftermath of the Ebola epidemic, which prompted large‐scale donor investment and health system rebuilding. Despite these efforts, inequities persist due to weak infrastructure, workforce shortages and uneven distribution of services, leading to wealth‐based disparities in SBA and ITN access. Furthermore, disrupted trust in formal health systems and persistent poverty among rural populations may limit uptake of available services, even when coverage expands [31, 49]. These patterns in both countries suggest that while national equity gaps endure, targeted and sustained interventions can improve health service utilization among the poor, particularly in areas like ANC and basic sanitation. Again, the observed rural advantage in Ghana's ANC coverage (94.2% rural vs. 86.0% urban) provides an empirical example of how targeted interventions work. This success could be largely attributed to the CHPS initiative, which shifted care from centralized hospitals to community clinics [50]. Similarly, the NHIS maternal exemption policy has demonstrably reduced financial barriers at the point of service for the poorest women, contributing to the pro‐poor equity observed in ANC visits.

Additionally, our CCI revealed a surprising outcome, with Liberia achieving a higher score of 77.9% compared to Ghana's 63.3%, despite Ghana having a more stable health system and stronger economic foundations. This unexpected result may be explained by the concentrated investments made in Liberia's health sector during the post‐crisis recovery period, particularly following the Ebola outbreak. These efforts reflect the impact that strong political commitment and international support can have in accelerating health system progress, even in settings with considerable fragility [31]. Nevertheless, Liberia's higher overall coverage conceals notable disparities, as access to many essential services remains unevenly distributed and tends to favour wealthier populations.

Moreover, we found urban–rural inequalities between both countries, which highlight systemic imbalances. In Ghana, the observed rural advantage in certain indicators, such as ANC, improved sanitation and exposure to health promotion, can be attributed to targeted interventions like the CHPS initiative, which has expanded basic healthcare delivery in rural settings [50, 51]. Conversely, Liberia presents a more complex pattern, where urban populations have better access to infrastructure‐dependent services such as drinking water and sanitation, whereas rural dwellers appear to benefit more from specific outreach programs introduced during post‐Ebola recovery efforts [30, 31]. These variations underscore the importance of designing health strategies that are sensitive to local geographic and infrastructural contexts. Without such tailored approaches, national efforts toward UHC risk entrenching spatial inequalities despite improvements in overall coverage [49, 52]. These findings affirm that UHC must be assessed not just by aggregate coverage but by its distribution across social groups, as emphasized by the WHO Consultative Group on Equity and UHC [6]. The data clearly show that progress toward SDG 3.8 is uneven and that expanding services without attention to who benefits may exacerbate health disparities.

## 5 Conclusion

This study examined health service coverage and inequalities in Ghana and Liberia using nationally representative DHS data. Despite moderate levels of coverage in both countries, substantial disparities persist, particularly along wealth and residential lines undermining progress toward equitable UHC. Ghana demonstrated relatively better coverage in preventive services such as ANC and improved water access, whereas Liberia showed marginally higher treatment coverage, likely reflecting recent post‐Ebola recovery investments. However, in both countries, pro‐rich inequities in access to SBA, ITN use and treatment for common illnesses remain widespread. These findings suggest that improving national averages alone is insufficient. A deliberate focus on addressing wealth‐ and location‐based disparities is essential to ensuring that UHC reforms are truly inclusive and equitable. Strengthening community‐based services, reducing financial barriers and targeting interventions to the most disadvantaged populations will be essential to accelerate progress toward SDG 3.8 in both settings.

### 5.1 Strengths and Limitations

A key strength of this study lies in its use of nationally representative DHS data, which are standardized across countries and allow for robust, population‐level comparisons. This enhances the reliability and generalizability of the findings to broader national contexts. Additionally, the study employed an equity‐focused analytical approach using both the SII and the RII, enabling a more nuanced understanding of disparities in health service access across wealth quintiles and residential settings. The comparative nature of the study, examining Ghana and Liberia side by side, also provides important insights into how differing policy environments and post‐crisis contexts influence health coverage outcomes. Furthermore, by incorporating a wide range of indicators from preventive interventions like ANC and ITN use to treatment indicators and composite indices, the study offers a comprehensive view of primary healthcare service delivery and equity.

However, several limitations should be acknowledged. The cross‐sectional nature of the data means that the study cannot establish causality or track trends over time. As a secondary data analysis, the scope of the study was limited to the variables collected in the DHS, potentially excluding other relevant factors such as quality of care, user satisfaction and informal barriers to access. In addition, the reliance on self‐reported data may introduce recall bias or social desirability bias, particularly in the reporting of service use. Moreover, we observed that some of our results had wide confidence interval, indicating reduced precision in our estimates reported. Therefore, caution should be exercised when interpreting the results. Finally, although the study identifies disparities in access, it does not fully capture deeper contextual or systemic drivers such as cultural norms, political factors, or subnational policy variations that may contribute to the observed inequalities.

## Author Contributions

*Conceptualization:* Samuel Asamoah, Michael Sarfo. *Methodology:* Samuel Asamoah, Michael Snowden and Jamie P. Halsall. *Formal analysis:* Samuel Asamoah, Michael Sarfo, Michael Snowden, Sandra Konadu Bonnah, Angwach Abrham Asnake and Olanrewaju Lawal. *Writing (initial draft):* Olanrewaju Lawal, Michael Snowden, Sandra Konadu Bonnah, Angwach Abrham Asnake and Michael Snowden. *Writing (revision and editing):* Samuel Asamoah, Michael Sarfo, Olanrewaju Lawal, Sandra Konadu Bonnah, Godness Kye Biney, Michael Snowden, Jamie P. Halsall and Angwach Abrham Asnake. *Supervision:* Michael Sarfo. All authors read and accepted the final version for publication. Michael Sarfo had the final responsibility to submit the work.

## Funding

The authors have nothing to report.

## Conflicts of Interest

The authors declare no conflicts of interest.

## Data Availability

All result‐based data are in the manuscript. In addition, the datasets can be accessed at http://goo.gl/ny8T6X.

## References

[1] World Health Organization , G. Carrin , C. James , and D. Evans , Achieving *U*niversal *H*ealth *C*overage: *D*eveloping *F*inancing *S*ystem (World Health Organization, 2005), https://iris.who.int/handle/10665/340519.

[2] World Health Organization , The World Health Report *2008: Primary Health Care: Now More Than Ever* (World Health Organization, 2008), http://www.who.int/whr/2008/en/.

[3] J. Kutzin , “Health Financing for Universal Coverage and Health System Performance: Concepts and Implications for Policy,” Bulletin of the World Health Organization 91, no. 8 (2013): 602–611. 23940408 10.2471/BLT.12.113985 PMC3738310

[4] World Health Organization . Making Fair Choices on the Path to Universal Health Coverage: Final Report of the WHO Consultative Group on Equity and Universal Health Coverage (World Health Organization, 2014), http://www.who.int/choice/documents/making_fair_choices/en/. 10.2471/BLT.14.139139 PMC4047814 24940009

[5] T. O'Connell , K. Rasanathan , and M. Chopra , “What Does Universal Health Coverage Mean?,” Lancet 383, no. 9913 (2014): 277–279. 23953765 10.1016/S0140-6736(13)60955-1

[6] O. F. Norheim , “Ethical Perspective: Five Unacceptable Trade‐Offs on the Path to Universal Health Coverage,” International Journal of Health Policy and Management 4, no. 11 (2015): 711–714. 26673330 10.15171/ijhpm.2015.184 PMC4629695

[7] C. A. Umeh and F. G. Feeley , “Inequitable Access to Health Care by the Poor in Community‐Based Health Insurance Programs: A Review of Studies From Low‐ and Middle‐Income Countries,” Global Health Science and Practice 5, no. 2 (2017): 299–314. 28655804 10.9745/GHSP-D-16-00286 PMC5487091

[8] A. P. Fenny , R. Yates , and R. Thompson , “Social Health Insurance Schemes in Africa Leave Out the Poor,” International Health 10, no. 1 (2018): 1–3. 29325056 10.1093/inthealth/ihx046

[9] World Health Organization , *World Health Statistics* 2023: *Monitoring Health for the SDGs, Sustainable Development Goals* (World Health Organization, 2023).

[10] United Nations. UN General Assembly , Transforming Our World: The 2030 Agenda for Sustainable Development (United Nations, 2015).

[11] G. Lagomarsino , A. Garabrant , A. Adyas , R. Muga , and N. Otoo , “Moving Towards Universal Health Coverage: Health Insurance Reforms in Nine Developing Countries in Africa and Asia,” Lancet 380, no. 9845 (2012): 933–943, 10.1016/S0140-6736(12)61147-7. 22959390

[12] World Health Organization , The World Health Report 2010: Health Systems Financing—The Path to Universal Coverage (World Health Organization, 2012). 10.2471/BLT.10.078741 PMC2878164 20539847

[13] P. Allotey , S. Verghis , F. Alvarez‐Castillo , and D. D. Reidpath , “Vulnerability, Equity and Universal Coverage: A Concept Note,” BMC Public Health [Electronic Resource] 12, no. S1 (2012): S2, 10.1186/1471-2458-12-S1-S2. 22992314 PMC3381707

[14] World Health Organization (WHO) and World Bank , WHO/World Bank Ministerial‐Level Meeting on Universal Health Coverage, 18–19 February 2013, WHO headquarters, Geneva, Switzerland (World Health Organization (WHO) and World Bank, 2013), https://www.who.int/news/item/12‐02‐2013‐who‐world‐bank‐convene‐ministerial‐meeting‐to‐discuss‐best‐practices‐for‐moving‐forward‐on‐universal‐health‐coverage.

[15] D. B. Evans , J. Hsu , and T. Boerma , “Universal Health Coverage and Universal Access,” Bulletin of the World Health Organization 91 (2013): 546–546A, 10.2471/BLT.13.125450. 23940398 PMC3738317

[16] E. C. Langat , P. Ward , H. Gesesew , and L. Mwanri , “Challenges and Opportunities of Universal Health Coverage in Africa: A Scoping Review,” International Journal of Environmental Research and Public Health 22, no. 1 (2025): 86–86. 39857539 10.3390/ijerph22010086 PMC11764768

[17] D. A. Adewole and K. O. Osungbade , “Nigeria National Health Insurance Scheme: A Highly Subsidized Health Care Program for a Privileged Few,” International Journal of TROPICAL DISEASE & Health 19 (2016): 1–11.

[18] E. Barasa , H. Ssekibubo , and P. A. Fugar , “The State of Universal Health Coverage in Africa,” Report of the Africa Health Agenda International Conference Commission. in Africa Health Agenda International Conference 2021 (Amref Health Africa, 2021).

[19] M. K. Chahed and C. Arfa , “Monitoring and Evaluating Progress Towards Universal Health Coverage in Tunisia,” PLOS Medicine 11, no. 9 (2014): e1001729. 25243673 10.1371/journal.pmed.1001729 PMC4170955

[20] K. Tinasti , “Morocco's Policy Choices to Achieve Universal Health Coverage,” Pan African Medical Journal 21, no. 53 (2015): 53. 26405489 10.11604/pamj.2015.21.53.6727 PMC4564433

[21] World Health Organization , Global Health Observatory—UHC Service Coverage Index (World Health Organization) (n.d). https://www.who.int/data/gho/indicator‐metadata‐registry/imr‐details/4834.

[22] R. K. Alhassan , E. Nketiah‐Amponsah , and D. K. Arhinful , “A Review of the National Health Insurance Scheme in Ghana: What Are the Sustainability Threats and Prospects?,” PLOS One 11, no. 11 (2016): e0165151. 27832082 10.1371/journal.pone.0165151 PMC5104458

[23] G. Humphreys , “Gabon Gets Everyone Under One Social Health Insurance Roof: Gabon's Comprehensive Health Insurance System Is Attracting Virtually All of Its Citizens, but to be Sustainable It Will Need to Get Costs Under Control,” Bulletin of the World Health Organization 91 (2013): 318–320. 23678193 10.2471/BLT.13.020513 PMC3646350

[24] I. A. Oyekola , J. O. Ojediran , O. A. Ajani , E. J. Oyeyipo , and B. Rasak , “Advancing Alternative Health Care Financing Through Effective Community Partnership: A Necessity for Universal Health Coverage in Nigeria,” Cogent Social Sciences 6, no. 1 (2020): 1776946.

[25] Ministry of Health , Kenya Universal Health Policy 2020–2030: Accelerating Attainment of Universal Health Coverage (Ministry ofHealth, 2021), http://guidelines.health.go.ke:8000/media/Kenya_Universal_Health_Coverage_Policy_2020__2030.pdf.

[26] C. Aantjes , T. Quinlan , and J. Bunders , “Towards Universal Health Coverage in Zambia: Impediments and Opportunities,” Development in Practice 26, no. 3 (2016): 298–307.

[27] World Health Organization , UHC Service Coverage Index (SDG 3.8.1) (World Health Organization) (n.d). https://platform.who.int/data/maternal‐newborn‐child‐adolescent‐ageing/indicator‐explorer‐new/MCA/coverage‐of‐essential‐health‐services‐(index‐interventions‐reproductive‐maternal‐newborn‐and‐child‐health)‐(sdg‐3.8.1).

[28] D. Cotlear , S. Nagpal , O. Smith , A. Tandon , and R. Cortez , Going Universal: How 24 Developing Countries Are Implementing Universal Health Coverage From the Bottom Up (World Bank Publications, 2015), https://www.worldbank.org/content/dam/Worldbank/document/HDN/Health/Annex‐6‐Going‐Universal.pdf.

[29] T. Ottersen , O. F. Norheim , World Health Organization Consultative Group on Equity and Universal Health Coverage , “Making Fair Choices on the Path to Universal Health Coverage,” Bulletin of the World Health Organization 92, no. 6 (2014): 389, 10.2471/BLT.14.139139. 24940009 PMC4047814

[30] M. E. Kruk , L. P. Freedman , G. A. Anglin , and R. J. Waldman , “Rebuilding Health Systems to Improve Health and Promote Statebuilding in Post‐Conflict Countries: A Theoretical Framework and Research Agenda,” Social Science & Medicine 70, no. 1 (2010): 89–97, 10.1016/j.socscimed.2009.09.042. 19850390

[31] L. Ako‐Egbe , R. Seifeldin , and S. Saikat , “Liberia Health System's Journey to Long‐Term Recovery and Resilience Post‐Ebola: A Case Study of an Exemplary Multi‐Year Collaboration,” Front Public Health 11 (2023): 1137865, 10.3389/fpubh.2023.1137865. 37404281 PMC10317185

[32] A. J. Barros , C. Ronsmans , and H. Axelson , “Equity in Maternal, Newborn, and Child Health Interventions in Countdown to 2015: A Retrospective Review of Survey Data From 54 Countries,” Lancet 379, no. 9822 (2012): 1225–1233, 10.1016/S0140-6736(12)60113-5. 22464386

[33] T. Boerma , C. AbouZahr , D. Evans , and T. Evans , “Monitoring Intervention Coverage in the Context of Universal Health Coverage,” PLOS Medicine 11, no. 9 (2014): e1001728, 10.1371/JOURNAL.PMED.1001728. 25243586 PMC4171108

[34] R. K. Alhassan , M. A. Antwi , and G. Sunkwa‐Mills , “Leveraging Local Health System Resources to Address Quality Healthcare Gaps in Sub‐Saharan African: Lessons From the SafeCare Quality Improvement Programme in Ghana,” BMC Health Services Research [Electronic Resource] 24, no. 1 (2024): 1499, 10.1186/s12913-024-11961-6. 39609663 PMC11603951

[35] M. Halter , V. Drennan , and K. Chattopadhyay , “The Contribution of Physician Assistants in Primary Care: A Systematic Review,” BMC Health Services Research [Electronic Resource] 13, no. 1 (2013): 223, 10.1186/1472-6963-13-223. 23773235 PMC3698179

[36] S. J. Ravi , C. M. Potter , L. Paina , and M. W. Merritt , “Post‐Epidemic Health System Recovery: A Comparative Case Study Analysis of Routine Immunization Programs in the Republics of Haiti and Liberia,” PLOS One 18, no. 10 (2023): e0292793, 10.1371/journal.pone.0292793. 37847680 PMC10581452

[37] K. Galactionova , T. A. Smith , D. de Savigny , and M. A. Penny , “State of Inequality in Malaria Intervention Coverage in Sub‐Saharan African Countries,” BMC Medicine [Electronic Resource] 15, no. 1 (2017): 185, 10.1186/s12916-017-0948-8. 29041940 PMC5646111

[38] S. T. Wafula , T. Habermann , and M. A. Franke , “What Are the Pathways Between Poverty and Malaria in Sub‐Saharan Africa? A Systematic Review of Mediation Studies,” Infectious Diseases of Poverty 12, no. 1 (2023): 58, 10.1186/s40249-023-01110-2. 37291664 PMC10249281

[39] A. E. Apeagyei , N. K. Patel , I. Cogswell , K. O'Rourke , G. Tsakalos , and J. Dieleman , “Examining Geographical Inequalities for Malaria Outcomes and Spending on Malaria in 40 Malaria‐Endemic Countries, 2010–2020,” Malaria Journal 23, no. 1 (2024): 206, 10.1186/s12936-024-05028-4. 38982498 PMC11234708

[40] D. Turkson and J. K. Ahiabor , “Implication of Natal Care and Maternity Leave on Child Morbidity: Evidence From Ghana,” Global Journal of Health Sciences 12, no. 9 (2020): 94, 10.5539/gjhs.v12n9p94.

[41] H. J. Oladipo , Y. A. Tajudeen , and I. O. Oladunjoye , “Increasing Challenges of Malaria Control in Sub‐Saharan Africa: Priorities for Public Health Research and Policymakers,” Annals of Medicine & Surgery 81 (2022): 104366, 10.1016/j.amsu.2022.104366. 36046715 PMC9421173

[42] World Health Organisation , WHO Traditional Medicine Strategy: 2014–2023 (World Health Organization, 2013) https://www.who.int/publications/i/item/9789241506096.

[43] K. B. Barimah , “Traditional Healers as Service Providers in Ghana's National Health Insurance Scheme: The Wrong Way Forward?,” Glob Public Health 8, no. 2 (2013): 202–208, 10.1080/17441692.2012.761262. 23336283

[44] A. Kpodotsi , E. A. Baku , J. H. Adams , and O. Alaba , “Socioeconomic Inequalities in Access and Use of Skilled Birth Attendants During Childbirth in Ghana: A Decomposition Analysis,” BMC Pregnancy Childbirth 21, no. 1 (2021): 850, 10.1186/s12884-021-04290-7. 34969366 PMC8719398

[45] J. Sumankuuro , J. Crockett , and S. Wang , “The Use of Antenatal Care in Two Rural Districts of Upper West Region, Ghana,” PLOS One 12, no. 9 (2017): e0185537, 10.1371/journal.pone.0185537. 28957422 PMC5619770

[46] A. A. Seidu , J. Okyere , E. Budu , H. O. Duah , and B. O. Ahinkorah , “Inequalities in Antenatal Care in Ghana, 1998–2014,” BMC Pregnancy Childbirth 22, no. 1 (2022): 478, 10.1186/s12884-022-04803-y. 35698085 PMC9190076

[47] J. Novignon , B. Ofori , K. G. Tabiri , and M. H. Pulok , “Socioeconomic Inequalities in Maternal Health Care Utilization in Ghana,” International Journal for Equity in Health 18, no. 1 (2019): 141, 10.1186/s12939-019-1043-x. 31488160 PMC6729067

[48] N. J. Blanchet , G. Fink , and I. Osei‐Akoto , “The Effect of Ghana's National Health Insurance Scheme on Health Care Utilisation,” Ghana Medical Journal 46, no. 2 (2012): 76–84. 22942455 PMC3426378

[49] A. Wagstaff , G. Flores , and J. Hsu , “Progress on Catastrophic Health Spending in 133 Countries: A Retrospective Observational Study,” Lancet Global Health 6, no. 2 (2018): e169–e179, 10.1016/S2214-109X(17)30429-1. 29248367

[50] J. K. Awoonor‐Williams , E. K. Sory , F. K. Nyonator , J. F. Phillips , C. Wang , and M. L. Schmitt , “Lessons Learned From Scaling Up a Community‐Based Health Program in the Upper East Region of Northern Ghana,” Global Health Science and Practice 1, no. 1 (2013): 117–133, 10.9745/GHSP-D-12-00012. 25276522 PMC4168550

[51] F. K. Nyonator , J. K. Awoonor‐Williams , J. F. Phillips , T. C. Jones , and R. A. Miller , “The Ghana Community‐Based Health Planning and Services Initiative for Scaling Up Service Delivery Innovation,” Health Policy and Planning 20, no. 1 (2005): 25–34, 10.1093/heapol/czi003. 15689427

[52] World Health Organization , WHO Global Strategy on *People‐Centred* and *Integrated Health Services*: *Interim Report* (World Health Organization, 2015). https://iris.who.int/handle/10665/155002.

